# Quantitative analysis of ureteral peristalsis in healthy women using transvaginal ultrasound combined with peristaltic analysis technology: a feasibility study

**DOI:** 10.3389/fbioe.2026.1862729

**Published:** 2026-08-04

**Authors:** Huihong Liu, Yuyu Zhou, Meifang Deng, Kaiju Luo, Yitong He, Shun Yang, Lvhuan Qiu, Qiuxiang Chen, Zhengyi Li

**Affiliations:** Department of Ultrasound, Shenzhen Second People’s Hospital, The First Affiliated Hospital of Shenzhen University Health Science Center, Shenzhen, China

**Keywords:** healthy women, peristaltic analysis, transvaginal ultrasonography, ureteral motility impairment, ureteral peristalsis

## Abstract

**Background:**

Abnormalities in ureteral motility often precede morphological changes, and early subtle lesions are easily overlooked, thereby increasing the risk of renal impairment. However, existing imaging assessments tend to focus on qualitative morphological analysis, and effective quantitative methods for assessing motility are still lacking.

**Methods:**

This single-center prospective observational study of healthy adult women (age range: 24–48 years) aimed to evaluate the feasibility of transvaginal ultrasound (TVUS) combined with peristaltic analysis (PA) for quantitative analysis of ureteral peristaltic waves. A total of 61 complete peristaltic waves from 30 ureters were recorded during 60-s dynamic acquisitions.

**Results:**

The study included 15 healthy women whose ureteral peristaltic waves exhibited a regular unidirectional propagation pattern from the proximal (renal) end to the distal (bladder) end. Based on Shapiro–Wilk tests combined with histograms and Q-Q plots, all five peristaltic parameters exhibited non-normal distributions. The M (Q1, Q3) values were as follows: transmission distance 0.39 cm (0.21, 0.71); transmission velocity 2.50 mm/s (1.20, 4.20); transmission time 1.59 s (1.19, 2.31); peristaltic intensity 1.55 arbitrary units (0.85, 2.64); and peristaltic interval 20.51 s (14.55, 26.75). Linear mixed model analysis showed that left-sided peristaltic intensity was significantly higher than right-sided intensity (*p* = 0.025), and bladder volume was negatively associated with transmission distance (β = −0.005, p = 0.029). No other significant side differences or associations were observed.

**Conclusion:**

This pilot study demonstrates that TVUS combined with PA is a feasible and non-invasive method for quantifying the dynamic characteristics of the pelvic segment of the ureter. Although these preliminary findings still need to be validated in larger pathological cohorts, this technique has exploratory potential for detecting early functional disorders associated with ureteral diseases.

## 1 Introduction

Ureteral peristalsis is a coordinated contraction of the smooth muscle in the wall, regulated by the precise control of the nervous system (Bohnenpoll and Kispert, 2014; Lang et al., 2002). Regular contractions not only facilitate the passage of urine but also prevent urine reflux, which is essential for maintaining the stability of the urinary system. Clinically, there are two main causes of abnormal ureteral motility, namely, inflammatory infiltration or fibrotic remodeling of the ureteral wall itself (Reicherz et al., 2023), and mechanical traction and fixation resulting from adhesions in the surrounding tissues (Seracchioli et al., 2007; Barra et al., 2018). Early or mild ureteral lesions may manifest solely as alterations in the neural microenvironment or as the effects of minor infiltration on the rhythm of smooth muscle contraction. The persistent impact of such subtle damage can exacerbate local inflammatory responses, thereby reducing the compliance and flexibility of the ureter and increasing the risk of irreversible long-term renal impairment (Kalayeh et al., 2024), eventually complicating the surgical intervention for the underlying condition (Lang et al., 2002). Development of assessment tools capable of sensitively capturing the early dynamic characteristics would be of significant value.

Currently, ideal quantitative tools for assessing ureteral motility are lacking. Although intravenous urography (IVU) is the “gold standard” for evaluating the ureteral function, it does not allow the quantitative analysis of key kinetic parameters, such as the frequency, wave velocity, and intensity of smooth muscle contractions (Nolte-Ernsting et al., 2003). Furthermore, IVU carries risks associated with radiation exposure and is limited by the need for intravenous administration of contrast agents (Muthusami and Ramesh, 2013). Magnetic resonance urography (MRU) is expensive, and its limited temporal resolution restricts its ability to capture long-term continuous dynamic images of peristaltic processes. Conventional transabdominal ultrasound can only provide a preliminary assessment of rhythmic movements through indirect observation of the “spray” sign. Transvaginal ultrasound (TVUS) scanning of the pelvic segment of the ureter, following the IDEA consensus guidelines, can achieve a visualization rate of over 90% (Guerriero et al., 2016); however, it is still limited to subjective visual assessment and is unable to achieve sub-micrometer tracking or objective quantification of minute tissue displacements. Consequently, a new, standardized assessment method capable of real-time quantification of ureteral peristaltic patterns is urgently required to identify early signals of the transition from physiological compensation to pathological instability in ureteral smooth muscle.

In recent years, peristaltic analysis (PA) technology, developed based on acoustic intelligence technology (AIT), has demonstrated excellent quantitative characterization capabilities in studies of the endometrium and gastric motility through the integration of sub-micrometer displacement tracking algorithms using high frame rate acquisition (approximately 50 Hz) (Zhang et al., 2022; Liao et al., 2022; Han et al., 2025). The physical basis of PA technology lies in capturing minute tissue displacements induced by smooth muscle contractions through high-frame-rate imaging combined with sub-micrometer displacement tracking algorithms (Kunz et al., 1998; Fanchin et al., 2000). Although Han et al. preliminarily validated the application of PA in gastric motility and obtained preliminary validation, its feasibility remains unconfirmed in the ureter—a structure characterized by a small luminal diameter and pronounced susceptibility to respiratory artifacts. Since impaired ureteral motility typically precedes macroscopic morphological alterations, capturing minute tissue displacements of the ureteral wall via high-frame-rate ultrasound holds great promise for the early quantitative assessment of ureteral peristaltic function.

Therefore, this study aims to investigate the feasibility of using PA technology to assess normal ureteral motility in healthy women, with the aim of providing preliminary reference values for future research on occult ureteral dysfunction.

## 2 Materials and methods

### 2.1 Study population

This study enrolled 15 healthy female volunteers aged 18–50 years between December 2025 and January 2026, collecting preliminary peristaltic wave data from 30 ureters. This human research study was approved by the Ethics Committee of Shenzhen Second People’s Hospital (Ethics Approval No. 2025-782-02  P J). The study was conducted in accordance with local regulations and institutional requirements, and written informed consent was obtained from all participants.

None of the participants had a history of pelvic congestion syndrome, urological diseases, renal dysfunction, or abdominal/pelvic surgery. To eliminate the confounding influence of autonomic nervous system imbalance on ureteral motility, individuals with hypertension, diabetes mellitus, spinal cord injury, or psychiatric disorders, such as anxiety, were excluded from the study.

### 2.2 Image acquisition and scanning procedure

Images were acquired using the Mindray A20 color Doppler diagnostic system equipped with a DE10-2WU transducer (frequency range: 3.0–9.0 MHz) and a PA kinetic analysis module. All procedures were conducted by a physician with over 15 years experience in gynecological ultrasound diagnosis.

To optimize imaging, subjects emptied their bladders, rested for 20 min, and were placed in the lithotomy position and instructed to relax the gluteal and abdominal muscles, allowing free transducer movement within the vaginal vault to visualize the entire length of the pelvic ureter (Guerriero et al., 2016). To eliminate any potential residual confounding factors, the study also calculated the post-void residual bladder volume for each participant and included it as a covariate in the statistical model. Bladder volume was calculated using the ellipsoid formula: length × width × height ×0.52. No special fasting was required, but examinations were avoided within 1 h after a meal to minimize gastrointestinal interference.

The study strictly adhered to the ureteral scanning method proposed by the International Deep Endometriosis Analysis (IDEA) group, using a transvaginal probe to localize the ureteral orifices in the bladder at the lateral fornix; the ureter was then continuously traced proximally along the lateral pelvic wall to the level of the iliac vascular bifurcation. During the scan, the target was confirmed by observing its characteristic long tubular hypoechoic structure and rhythmic smooth muscle peristalsis, to assess the ureter’s course and kinetic status (Guerriero et al., 2016).

To minimize respiratory and bowel artifacts during 60-s scans, we implemented the following measures. Because female subjects predominantly use thoracic breathing (Kaneko and Horie, 2012) and deep pelvic displacement due to diaphragm motion is typically ≤5 mm (Schwarz and Leach, 2000), respiratory impact on the ureteral anterior wall was limited. The transducer was fixed at the vaginal fornix after identifying the optimal plane. If the real-time image did not meet the quality criteria, the recording was repeated immediately. For example,: (1) Sudden intestinal peristalsis causing displacement of the ureter or loss of the target cross-section; (2) Intestinal gas producing acoustic shadows or reverberation, obscuring the anterior wall of ureter (Han et al., 2025).

Dynamic scanning was performed in the PA mode. Referring to relevant studies on the peristaltic frequency of the ureter in healthy women (mean 3.5 times/min, range 2–10 times/min) (Lang et al., 2002), the duration of each dynamic recording in this study was set to 60 s. This duration ensured the capture of multiple complete peristaltic cycles and formed the basis for the precise quantification of the propagation velocity, frequency, and peristaltic wave intervals.

**FIGURE 1:**
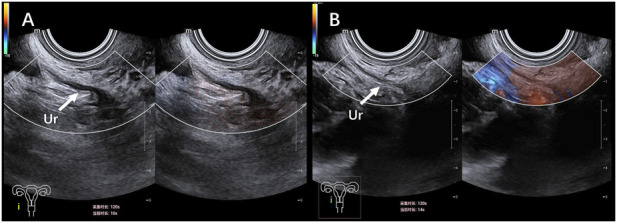
Visualization of ureteral peristalsis using the PA technique’s color-coded mode and vector arrow mode: **(A)** shows the interface of the PA technique’s vector arrow mode, and **(B)** shows the interface of the PA technique’s color-coded mode.

The study employed a combination of two visualization modes, the vector arrow mode and color-coded mode, to characterize the ureteral peristaltic waves in multiple dimensions (Figure 1); the vector map mode provided a detailed display of the instantaneous movement trajectories of the smooth muscle in the tube wall. The displacement path and amplitude of the ureter were assessed in real time by observing the spatial orientation and changes in the lengths of the vector arrows. The dynamic distribution and luminance pulsations of the arrows provided an intuitive representation of the velocity gradients and degree of contraction during the peristaltic process. The color-coded mode primarily illustrated the vertical displacement characteristics of the tubular walls. By capturing the spatiotemporal variations in color pixel signals, the propagation rhythm of the peristaltic wave could be accurately determined, whereas instantaneous changes in color saturation served as a visual reference for assessing the intensity of smooth muscle contraction.

### 2.3 Postprocessing of images and quantitative analysis

During post-processing, quantitative analysis of the recorded 60-s images was performed using PA technology. A blinded operator selected the most stable frame from each 60-s clip defined by the clearest wall definition, the most consistent spatiotemporal signal, and the fewest motion induced artifacts to place the ROI. The ROI was drawn from the ureterovesical junction to the iliac bifurcation (or maximal visible extent), covering the full anterior wall (the posterior wall is prone to cervical/bowel artifacts), and was then applied to the entire 60-s clip for full-length analysis.

The PA software tracked sub-micrometer-level displacements within the ROI based on high-frame-rate images, automatically generating a visualized spatiotemporal propagation map and outputting the following metrics: transmission distance (cm), transmission velocity (mm/s), transmission time (s), peristaltic interval (s), peristaltic intensity (AU), and propagation direction (unidirectional/reverse). Peristaltic intensity was defined as the root mean square amplitude of tissue displacement within the ROI, normalized by the ultrasound system. Although expressed in arbitrary units (AU), the values are comparable across measurements because the same machine and identical settings were used.

### 2.4 Statistical analysis

Following image acquisition, a physician with over 15 years of experience in gynecological ultrasound diagnosis extracted the peristaltic wave parameters from the marker maps generated by the PA. All statistical analyses were performed using SPSS. Normality was assessed using the Shapiro–Wilk test, supplemented by histograms and Q-Q plots; parameters following a normal distribution are described as X ± S (SD), while those not following a normal distribution are provided as M (Q1, Q3). Due to the hierarchical structure of the data (multiple peristaltic waves per ureteral side, and bilateral ureters for each subject), a linear mixed model (LMM) was used to account for the non-independence of data within the same subject. Subject ID was treated as a random intercept, and fixed effects included side (left vs. right), parity, age, BMI, and bladder capacity. The dependent variables were various peristaltic parameters (propagation distance, velocity, time, intensity, and interval). LMM results were reported as estimates, standard errors, degrees of freedom, t-values, p-values, and 95% confidence intervals for fixed effects. To further assess the potential effect of bladder volume on the results, a reduced model was constructed by excluding bladder volume from the fixed effects while keeping all other aspects identical to the full model. All tests were two-sided, and *p* < 0.05 was considered statistically significant.

To assess measurement reproducibility, peristaltic wave data from all 15 subjects were reanalyzed by the same operator 1 month later (intra-observer reproducibility) and independently analyzed by a second operator (inter-observer reproducibility). Intraclass correlation coefficients (ICC) were calculated using a two-way mixed-effects model.

Given the exploratory nature of this pilot study, no adjustments for multiple comparisons were performed.

**FIGURE 2:**
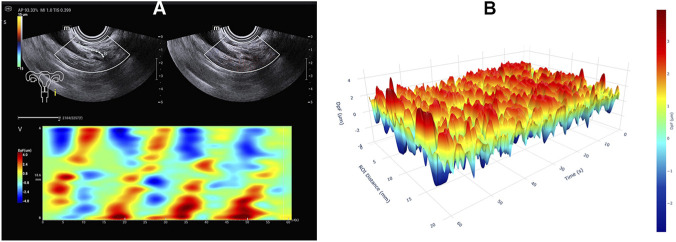
**(A)** Spatiotemporal propagation map of ureteral peristalsis generated by the PA system. The upper images show raw ultrasound and color-mode acquisition of ureteral wall motion. The lower heatmap displays alternating red-blue oblique waveforms representing vertical tissue displacement during peristaltic propagation. Red indicates movement away from the probe (positive velocity), and blue indicates movement toward the probe (negative velocity). The diagonal orientation of the wave bands reflects wave propagation from proximal to distal ureteral over time. The vertical axis (V) corresponds to tissue displacement velocity, while the horizontal axis (T) represents time. This visualization illustrates continuous wave propagation without retrograde events. **(B)** Three-dimensional reconstruction of the peristaltic propagation map shown in panel **(A)**. The X-axis represents time (s), the Y-axis represents spatial position along the ROI (mm), and the Z-axis shows vertical displacement amplitude (DpF, μm). The color scale is identical to that used in the 2D propagation map, facilitating direct comparison. This 3D visualization provides an intuitive representation of wave amplitude, periodicity, and propagation patterns.

Following real-time image acquisition, the PA system constructed spatiotemporal propagation maps to characterize the dynamic behavior of the ureteral wall throughout the entire scanning duration. The resulting image displayed characteristic alternating red and blue band patterns. Red regions indicated tissue motion away from the probe (positive velocity), while blue regions reflected motion toward the probe (negative velocity). The intensity of each color corresponded to the magnitude of vertical velocity, representing the strength of peristalsis. The steeper the diagonal pattern, the greater the propagation speed of the peristaltic wave. The orientation of these diagonal bands reflected the direction of propagation, for example, the presence of multiple red-to-blue slanted streaks from the lower right to the upper left indicated that the peristaltic wave propagated from the proximal (renal) to the distal (bladder) end (anterograde peristalsis), which is consistent with normal physiological rhythm (Figure 2A).

To enable the quantitative analysis of peristaltic dynamics, the extracted ureteral wall displacement vector data were imported into a Python-based analysis framework, successfully constructing propagation maps that reflected the temporal and spatial evolution of peristaltic waves. In this 3D rendering heatmap, the X-axis represents the duration of continuous observation, the Y-axis represents the ROI Distance along the longitudinal axis of the ureter, and the Z-axis represents wall displacement (DpF) (Figure 2B).

## 3 Results

This study utilized TVUS combined with high-frame-rate ultrasound PA to capture real-time visualization of the peristaltic process in the bilateral ureters (30 ureters in total) of 15 participants. The participants had a mean age of 37.33 ± 7.12 years (range: 24–48 years) and a mean BMI of 21.50 ± 2.80 kg/m^2^. Approximately 46.7% (7/15) were nulliparous and 53.3% (8/15) were parous. The median bladder volume was 43.9 mL (IQR: 27.6–65 mL), showing considerable inter-individual variability.

All 30 ureters exhibited a consistent unidirectional pattern of propagation from the proximal (renal) end to the distal (bladder) end (anterograde peristalsis), with no retrograde peristalsis observed.

**FIGURE 3:**
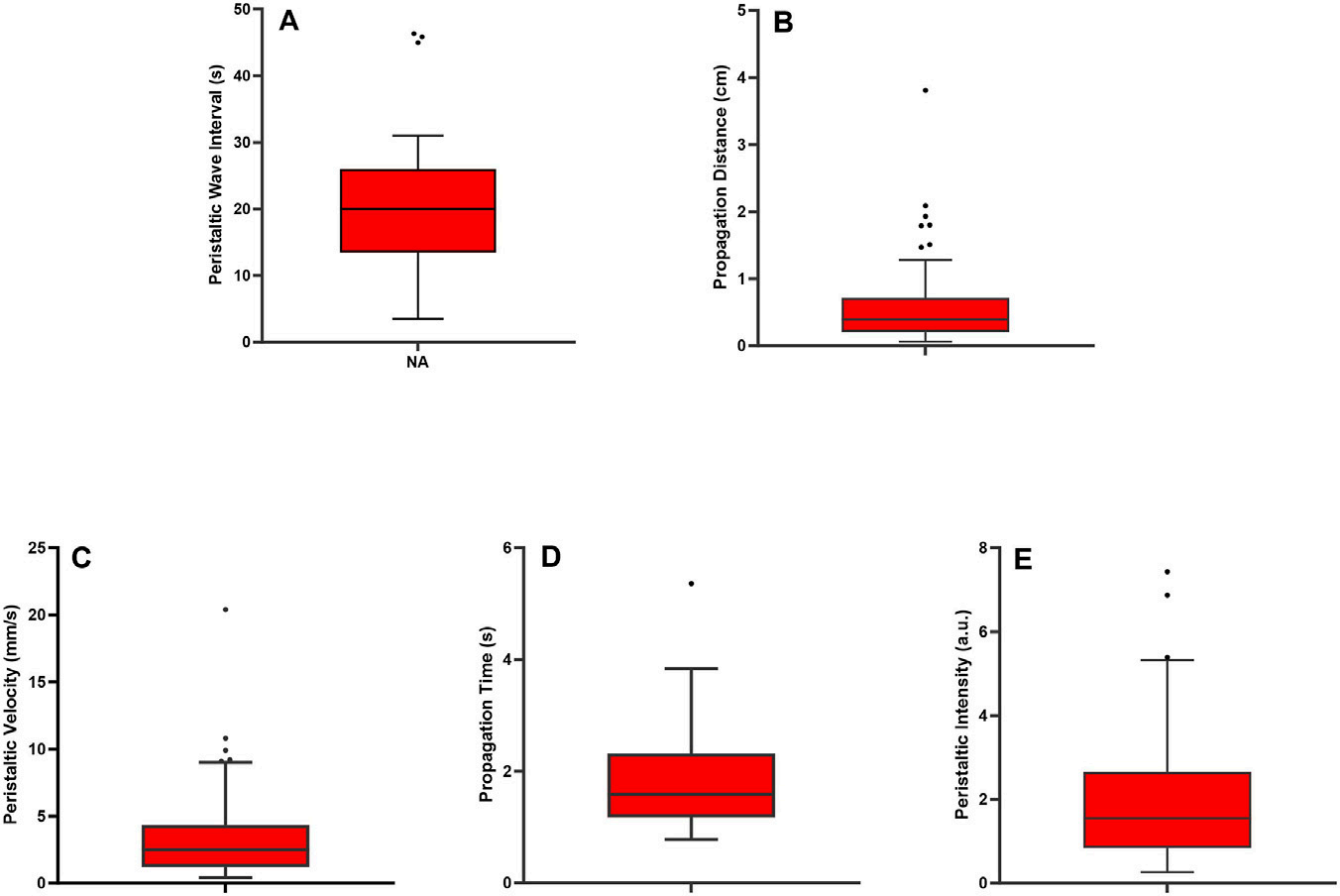
Box plots of peristaltic parameters in the ureters of healthy women, measured using TVUS combined with PA technology: **(A)** peristaltic wave interval; **(B)** distance; **(C)** velocity; **(D)** transit time; **(E)** intensity.

A total of 61 complete peristaltic waves were recorded from 30 ureters. During the 60-s acquisition, the number of waves per ureter ranged from 1 to 5 on the left side (median 2) and from 1 to 3 on the right side (median 2). Normality was assessed using Shapiro–Wilk tests combined with histograms and Q-Q plots, which revealed that all five peristaltic parameters exhibited skewed distributions (Table 1; Figure 3). Therefore, the data are presented as [M (Q1, Q3)]: transmission distance, 0.39 cm (0.21.0.71); transmission velocity, 2.50 mm/s (1.20.4.20); transmission time, 1.59 s (1.19.2.31); peristaltic intensity, 1.55 AU (0.85.2.64); and peristaltic interval, 20.51 s (14.55.26.75) (Table 1; Figure 3).

**TABLE 1:** Statistical summary of peristaltic wave dynamics.

Characteristics	Value
Intensity (AU)	1.55 (0.85, 2.64)
Velocity (mm/s)	2.50 (1.20, 4.20)
Distance (cm)	0.39 (0.21, 0.71)
Time (s)	1.59 (1.19, 2.31)
Interval (s)	20.51 (14.55, 26.75)

Data are expressed as Median (Q1, Q3) for skewed variables. AU: arbitrary units.

**FIGURE 4:**
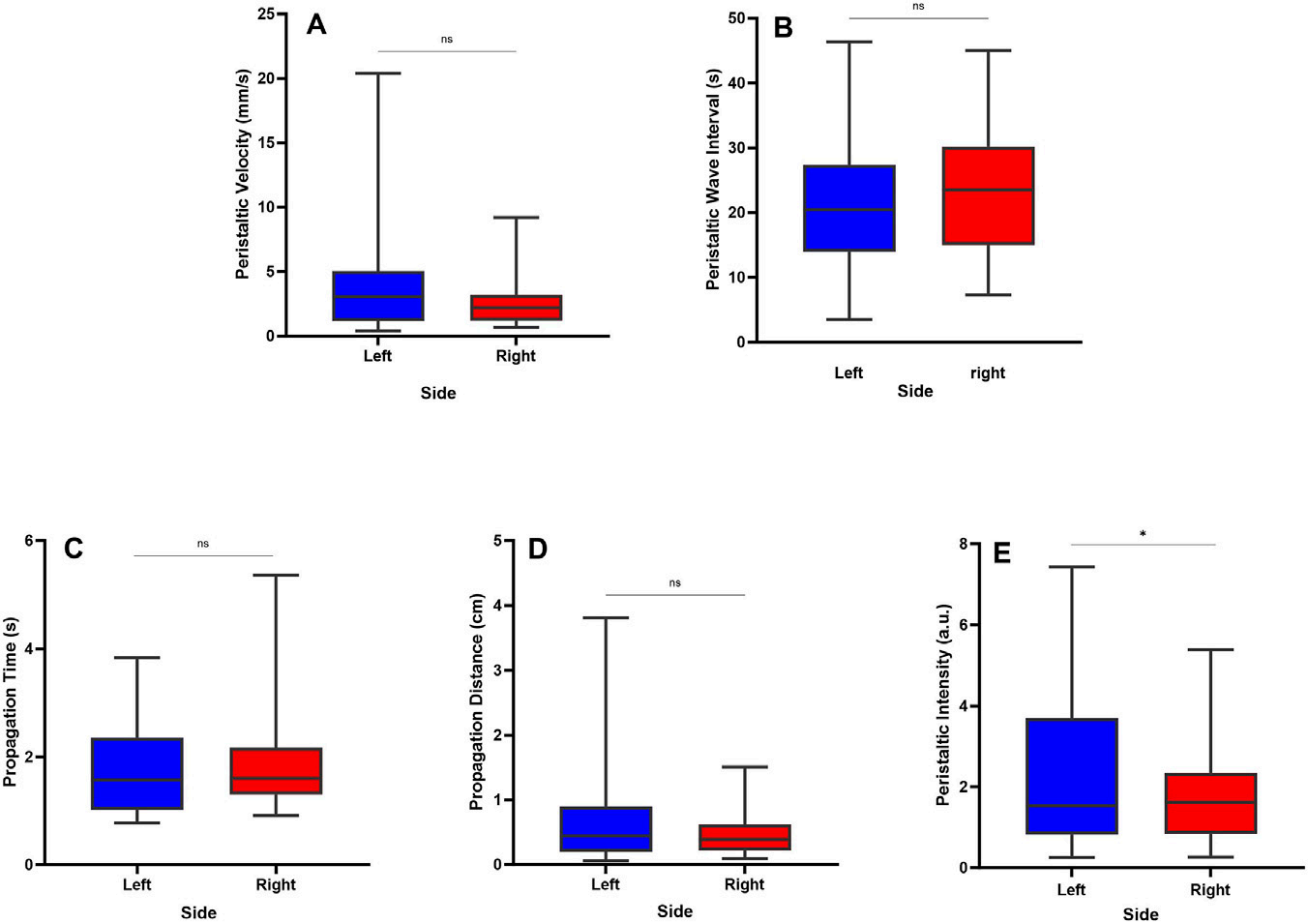
Comparison of bilateral ureteral peristaltic parameters: **(A)** velocity; **(B)** interval; **(C)** transit time; **(D)** distance; **(E)** intensity; indicates *p* < 0.05, n. s. Indicates *p* > 0.05.

The analysis results showed that ureteral peristalsis intensity on the left side was significantly higher than that on the right (β = 0.648, 95% CI: 0.086 to 1.211, *p* = 0.025), and bladder volume was negatively associated with propagation distance (β = −0.005, 95% CI: −0.010 to −0.001), *p* = 0.029). Age, body mass index, and parity showed no statistically significant association with the other peristaltic parameters (all p > 0.05) (Table 2; Figure 4). Sensitivity analysis excluding bladder volume yielded consistent results: left-sided peristaltic intensity remained significantly higher (β = 0.645, 95% Cl: 0.083 to 1.206, p = 0.025), and other clinical factors remained non-significant (Table 3).

**TABLE 2:** Results of the linear mixed model analysis (model 1).

Characteristics	Variable	F	*p*	β (95% CI)
Intensity (AU) (n = 61)	Side	5.38	0.025*	0.63 (0.07.1.21)
​	Parity	0.38	0.550	−0.74 (-3.43, 1.94)
​	BMI (kg/m^2^)	0.70	0.422	−0.10 (-0.37, 0.17)
​	Age (years)	0.16	0.697	−0.03 (-0.21, 0.14)
​	Bladder volume (ml)	0.09	0.766	−0.006 (-0.05, 0.04)
Velocity (mm/s) (n = 61)	Side	0.24	0.624	0.40 (-1.22, 2.02)
​	Parity	0.47	0.51	1.33 (-3.03, 5.69)
​	BMI (kg/m^2^)	0.02	0.899	−0.03 (-0.47, 0.42)
​	Age (years)	1.48	0.252	0.16 (-0.13, 0.44)
​	Bladder volume (ml)	0.08	0.79	0.01 (-0.08, 0.06)
Distance (cm) (n = 61)	Side	0.38	0.539	0.05 (-0.12, 0.22)
​	Parity	4.33	0.066	0.26 (-0.12, 0.22)
​	BMI (kg/m^2^)	0.12	0.735	0.26 (-0.02.0.53)
​	Age (years)	0.16	0.702	−0.004 (0.03, 0.02)
​	Bladder volume (ml)	6.47	0.029*	−0.005 (-0.01, −0.001)
Time (s) (n = 61)	Side	0.27	0.604	−0.12 (-0.56, 0.33)
​	Parity	0.24	0.631	0.21 (-0.73, 1.15)
​	BMI (kg/m^2^)	0.44	0.519	−0.03 (-0.13, 0.07)
​	Age (years)	0.52	0.484	0.02 (-0.04, 0.08)
​	Bladder volume (ml)	0.14	0.716	−0.003 (-0.02, 0.01)
Interval (s) (n = 61)	Side	0.44	0.515	−2.48 (-10.19, 5.22)
​	Parity	0.99	0.328	5.55 (-5.85, 16.94)
​	BMI (kg/m^2^)	0.77	0.387	0.47 (-0.63, 1.58)
​	Age (years)	0.01	0.916	−0.04 (-0.75, 0.68)
​	Bladder volume (ml)	1.55	0.224	0.12 (-0.07, 0.31)

Abbreviations: CI: confidence interval; BMI: body mass index.

Model 1 used an LMM, to incorporate age; BMI, side, parity, and bladder capacity to analyze their effects on peristaltic parameters, with subject ID, as the random intercept and the right side as the reference group; for parity, nulliparous women served as the reference group. *indicates statistically significant results (p < 0.05).

**TABLE 3:** Results of the linear mixed-effects model analysis (Model 2).

Characteristics	Variable	F	*p*	β (95% CI)
Intensity (AU) (n = 61)	Side	5.34	0.025*	0.65 (0.08, 1.20)
​	Parity	0.38	0.551	−0.70 (-3.23, 1.82)
​	BMI (kg/m^2^)	0.82	0.384	−0.11 (-0.36, 0.15)
​	Age (years)	0.14	0.716	−0.03 (-0.19, 0.14)
Velocity (mm/s) (n = 61)	Side	0.25	0.618	0.40 (-1.21, 2.06)
​	Parity	0.57	0.468	1.39 (-2.70, 5.48)
​	BMI (kg/m^2^)	0.03	0.865	−0.03 (-0.45, 0.39)
​	Age (years)	1.84	0.204	0.16 (-0.10, 0.42)
Distance (cm) (n = 61)	Side	0.26	0.611	0.04 (-0.13, 0.21)
​	Parity	3.58	0.087	0.29 (-0.05, 0.62)
​	BMI (kg/m^2^)	0.2	0.664	−0.007 (-0.04, 0.03)
​	Age (years)	0.71	0.422	0.008 (-0.01, 0.03)
Time (s) (n = 61)	Side	0.28	0.597	−0.12 (-0.56, 0.33)
​	Parity	0.31	0.589	0.23 (-0.66, 1.11)
​	BMI(kg/m^2^)	0.54	0.478	−0.03 (-0.12, 0.06)
​	Age (years)	0.76	0.402	0.02 (-0.03, 0.08)
Interval (s) (n = 61)	Side	0.33	0.572	−2.17 (-9.91, 5.57)
​	Parity	0.67	0.42	4.55 (-6.81, 15.91)
​	BMI (kg/m^2^)	1.09	0.304	0.56 (-0.54, 1.67)
​	Age (years)	0.40	0.533	−0.21 (-0.87, 0.46)

Abbreviations: CI: confidence interval; BMI: body mass index.

Model 2 used an LMM, to incorporate age; BMI, side, and parity to analyze their effects on peristaltic parameters, with subject ID, as the random intercept, the right side as the reference group for side, and nulliparity as the reference group for parity. *indicates statistically significant results (p < 0.05).

The intraclass correlation coefficient (ICC) ranged from 0.759 to 0.848 for intra-observer evaluation and from 0.780 to 0.843 for interobserver evaluation. All p-values were <0.001, indicating that this measurement parameter has good to excellent reproducibility (Supplementary Tables S1-S2).

Analysis of the 3D plot identified rhythmic peak waveforms characterized by organized architecture, confirming the presence of intact ureteral motility and highly uniform peristaltic directionality in the subject (Figure 2B).

## 4 Discussion

This preliminary study suggests that TVUS combined with PA technology can assess the dynamic characteristics of the pelvic ureter in healthy women. Unlike traditional imaging modalities (intravenous urogram, MRU, and transabdominal ultrasound), which provide only indirect or qualitative information, PA enables real-time, quantitative acquisition of ureteral peristaltic parameters using submicrometer displacement tracking (Nolte-Ernsting et al., 2003; Muthusami and Ramesh, 2013). The median peristaltic interval measured in this study (20.51 s, approximately 2.9 times/min) is consistent with the normal range reported in *Campbell and Walsh’s Urology* (2–6 times/min) and with radionuclide data (<4 times/min; Wemyss-Holden et al., 1993; Partin et al., 2021). Based on the confirmation of this frequency, PA can also capture parameters such as distance, velocity, time, intensity, and propagation direction, providing a multidimensional functional profile. These parameters reflect the compliance and elasticity of the ureteral wall. Abnormalities in these parameters may indicate increased wall stiffness or reduced elasticity. This highlights the potential of this PA technology for screening early functional impairment in conditions such as ureteral fibrosis and diseases characterized by wall sclerosis (e.g., ureteral endometriosis). As emphasized by Saadeh et al. (2024), this condition typically remains asymptomatic until severe strictures or renal dysfunction develop. Theoretically, PA technology could identify subtle dynamic abnormalities before conventional imaging reveals obvious structural obstruction. However, this application remains hypothetical and requires rigorous validation in pathological cohorts (Saadeh et al., 2024).

The first finding of this study is that all five peristaltic parameters exhibited non-normal distributions. This may be due to the presence of clear lower limits for each parameter: the refractory period following the action potential (>10 s) limits the interval from shrinking indefinitely, thereby preventing tetanic spasms (Burdyga and Wray, 2005); intensities below the threshold fail to close the lumen; velocity and distance are constrained by the compliance of the ureter wall, with excessively slow or short movements leading to failure to propel the contents (Kalayeh et al., 2024); and duration cannot be infinitely shortened due to anatomical length limitations. At the same time, the upper limits of these parameters are adjustable: as urine volume increases, the pacemaker frequency accelerates and the interval shortens; the stretching of the ureter wall enhances contraction through mechanosensitive channels (L-type calcium channels, IP_3_ receptors), thereby increasing intensity, velocity, and distance while shortening duration (Constantinou et al., 1977; Amedzrovi Agbesi et al., 2026). This “lower-bound constrained, upper-bound flexible” pattern results in a skewed distribution. However, this interpretation remains speculative and should be considered hypothesis generating, requiring further experimental validation.

The second finding was that peristaltic intensity was significantly higher on the left side than on the right, while other parameters were symmetrical. This may stem from anatomical differences between the two sides of the pelvis: on the left side, the ureter lies in close proximity to the descending and sigmoid colon (Azimuddin et al., 1999), where high-amplitude propulsive contractions generate higher local pressure, whereas the contents of the cecum on the right side are liquid and exert lower pressure. The left side, which is chronically exposed to higher extraluminal pressure, may compensate through a mechanosensitive response of the smooth muscle–that is, external forces trigger positive-feedback contractions mediated by L-type calcium channels and IP3 receptors, causing the left side to spontaneously maintain higher intensity to counteract resistance (Amedzrovi Agbesi et al., 2026). This suggests that when evaluating pelvic pathologies (such as deep endometriosis), the baseline physiological increase in left-sided tone must be considered to avoid misinterpreting bilateral function.

The third finding was that bladder volume was negatively associated with propagation distance and was independent of other parameters. This study speculates that this is related to increased distal ureteral resistance caused by bladder distension. Kalayeh et al. found that elevated bladder pressure increases the minimum amplitude required to prevent peristaltic reflux and exacerbates reflux flow (Kalayeh et al., 2024). Based on this, it is hypothesized that as bladder volume increases, distal resistance rises, causing peristaltic waves to terminate prematurely before reaching the ureteral orifice, resulting in a shorter travel distance. In contrast, velocity, amplitude, and interval are likely determined primarily by proximal pacemakers and the intrinsic properties of smooth muscle (Lang et al., 2002; Lang et al., 1998) and are insensitive to downstream resistance, hence showing no significant association. Although the shortened distance should have reduced the time, no significant change in time was observed, possibly due to small effect sizes or data variability. These results suggest that bladder volume may primarily affect spatial constraints rather than rhythm or contractile capacity. Given the small sample size and the narrow physiological range of bladder volumes, this negative association may be a chance finding and requires validation with a larger sample.

In this small cohort of healthy women, we did not detect significant association between age, BMI, obstetric history, and any of the ureteral peristaltic parameters. Larger studies are needed to determine whether these baseline factors truly have no influence on ureteral motility.

## 5 Limitations

The current study has certain limitations. First, the sample size was small (15 healthy women of childbearing age), and the study did not include postmenopausal women or patients with pelvic diseases, limiting statistical power and external validity. Second, menstrual cycle phase was not recorded. As per standard practice for transvaginal ultrasound, all scans were performed outside the bleeding phase, but estrogen and progesterone levels still fluctuate across the follicular and luteal phases and may modulate ureteral smooth muscle contractility (Lim et al., 2021). Thus, the potential confounding effect of cycle phase on peristaltic parameters cannot be completely excluded. Future studies should include larger and more diverse populations, record specific cycle phases (e.g., follicular vs. luteal), use multiple operators, and conduct longitudinal follow-ups to validate and extend our findings.

## 6 Conclusion

This pilot study demonstrated the feasibility of using TVUS combined with PA technology to quantify ureteral peristalsis. Given the small sample size, these preliminary physiological findings require rigorous validation in larger cohorts—particularly in patients with urological conditions—to establish their clinical utility.

## Data Availability

The original contributions presented in the study are included in the article/Supplementary Material, further inquiries can be directed to the corresponding authors.

## Supplementary material

SUPPLEMENTARY TABLES S1–S2 Intra- and inter-observer reproducibility of peristaltic parameters.
