# Supplementary material for: Quantitative analysis of ureteral peristalsis in healthy women using transvaginal ultrasound combined with peristaltic analysis technology: a feasibility study
Source: Front Bioeng Biotechnol. 2026 Aug 4;14:1862729. doi: 10.3389/fbioe.2026.1862729 (PMC13481499; doi:10.3389/fbioe.2026.1862729)
Supplement: Supplementary file 1 [file Table1.docx]

**Supplementary Table 1**

**Intra observer Reproducibility Results**

| Parameter | ICC | 95% CI | *p* |
| --- | --- | --- | --- |
| Intensity (AU) | 0.821 | 0.623 - 0.920 | < 0.001 |
| Distance | 0.759 | 0.512 - 0.890 | < 0.001 |
| Time(s) | 0.832 | 0.644 - 0.925 | < 0.001 |
| Velocity(cm/s) | 0.848 | 0.675 - 0.933 | < 0.001 |
| Interval(s) | 0.822 | 0.466 - 0.949 | < 0.001 |

**Supplementary Table 2**

**Inter observer Reproducibility Results**

| Parameter | ICC | 95% CI | *p* |
| --- | --- | --- | --- |
| Intensity(AU) | 0.824 | 0.629 - 0.921 | < 0.001 |
| Distance | 0.780 | 0.549 - 0.901 | < 0.001 |
| Time(s) | 0.786 | 0.560 - 0.903 | < 0.001 |
| Velocity(cm/s) | 0.843 | 0.666 - 0.930 | < 0.001 |
| Interval(s) | 0.829 | 0.484 - 0.951 | < 0.001 |
